# Paraneoplastic Trichomegaly as a Harbinger of Metastatic Renal Cell Carcinoma: A Case Report

**DOI:** 10.7759/cureus.83550

**Published:** 2025-05-06

**Authors:** Allison Reichl, Rena Callahan

**Affiliations:** 1 Internal Medicine, University of California Los Angeles, Los Angeles, USA; 2 Hematology and Oncology, University of California Los Angeles, Los Angeles, USA

**Keywords:** eyelash trichomegaly, hypertrichosis, metastatic renal cell carcinoma, paraneoplastic dermatoses, paraneoplastic syndromes

## Abstract

Renal cell carcinoma (RCC) is a common malignancy, often diagnosed incidentally on cross-sectional imaging. While classic symptoms such as flank pain, hematuria, and a palpable abdominal mass are well-known, RCC may present with more insidious findings. Here, we describe a case of paraneoplastic trichomegaly as the initial manifestation of metastatic RCC. A man in his 60s presented with progressive eyelash lengthening and mild hypertrichosis of the extremities and beard. Medication review and initial endocrine workup were unrevealing. Laboratory tests showed iron deficiency anemia, and subclinical hematuria was noted on urinalysis. Imaging revealed a right renal mass and retroperitoneal lymphadenopathy. Biopsy confirmed clear cell RCC. The patient was treated with neoadjuvant immunotherapy followed by radical nephrectomy. His trichomegaly improved with treatment. Unfortunately, he experienced disease progression and passed away 19 months later due to thrombotic complications of metastatic RCC. Trichomegaly is a dermatologic finding typically associated with congenital syndromes, drug effects, or HIV. Its paraneoplastic occurrence is exceptionally rare, and the molecular mechanisms driving paraneoplastic hypertrichosis remain unclear. This case emphasizes that occult malignancy should be considered in the setting of unusual mucocutaneous findings.

## Introduction

Renal cell carcinoma (RCC) is a relatively common malignancy, with a peak incidence between 60 and 70 years of age; its lifetime prevalence is 2.3% in men and 1.3% in women [1,2]. The majority of RCCs are identified incidentally, in part due to the increasing availability of cross-sectional abdominal imaging techniques [3]. At the time of RCC diagnosis, 70% of cases are stage I and 11% are Stage IV [4]. The three most common histological subtypes of RCC are clear cell (75-80%), papillary (10-15%), and chromophobe (5%) [5].

The classically taught triad of flank pain, palpable abdominal mass, and hematuria occurs in less than 10% of patients with newly diagnosed RCC [6]. Of patients with RCC, 10-40% have associated paraneoplastic syndromes (PNS), which sometimes are the presenting complaint. These resolve with nephrectomy in up to 52% of cases [7]. Here we present an unusual case of paraneoplastic eyelash growth associated with the onset of RCC.

## Case presentation

**Figure 1:**
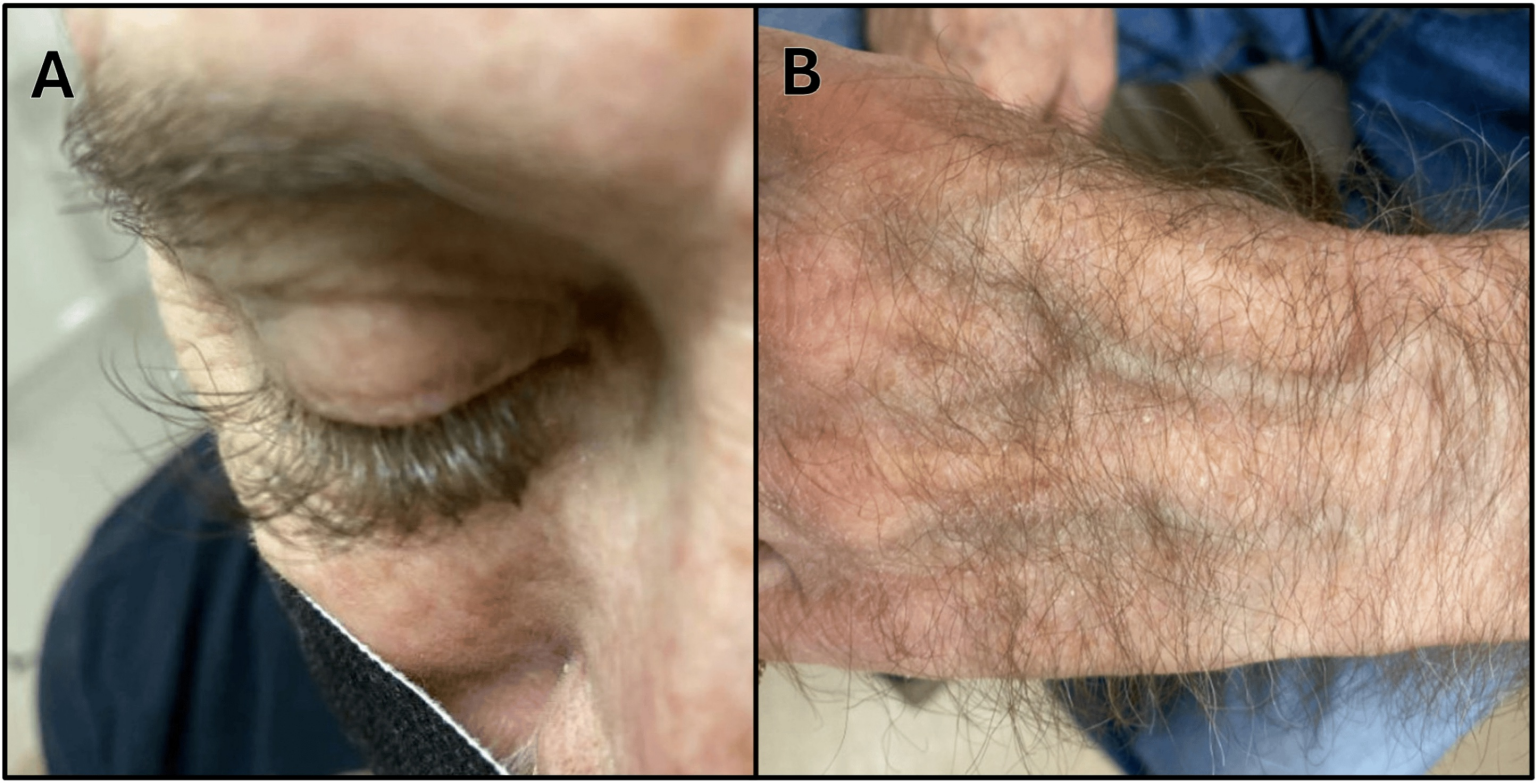
Patient's (A) lengthened eyelashes and (B) left hand hair growth.

A man in his 60s presented to his primary care physician for several months of unusual lengthening and thickening of his eyelashes (Figure 1A). On further history, he also endorsed mild lengthening of the hair on his arms, hands, beard, and lower extremities (Figure 1B). He was otherwise asymptomatic and not taking medications known to cause trichomegaly. Laboratory workup (Table 1) for endocrinopathies was benign. He was noted to have a mild normocytic anemia with low serum iron and low iron saturation. Ferritin was elevated. Urinalysis demonstrated trace blood. In hindsight, the patient recalled that he had experienced one episode of gross hematuria six months prior, which had resolved on its own.

**Table 1:** Initial Laboratory Results WBC: White Blood Cell; TIBC: Total Iron Binding Capacity; DHEA: Dehydroepiandrosterone Sulfate; FSH: Follicle Stimulating Hormone; TSH: Thyroid Stimulating Hormone

Test	Patient Value	Reference Range	Units
WBC Count	8.0	3.8-10.8	x10^3^/uL
Hemoglobin	12.1	13.2-17.1	g/dL
Platelet Count	362	140-400	x10^3^/uL
Iron	29	50-160	mcg/dL
TIBC	259	230-425	mcg/dL
Ferritin	563	24-380	ng/mL
Vitamin B12	534	200-1100	pg/mL
Folate	12.3	>5.4	ng/mL
Testosterone, Total	573	200-1100	ng/dL
Testosterone, Free	75	35-155	pg/mL
DHEA	88	22-244	mcg/dL
FSH	5.9	1.6-8.0	mIU/mL
Prolactin	6.7	2-18	ng/mL
TSH	1.37	0.4-4.5	mIU/L
Free T4	1.1	0.8-1.8	ng/mL
Hemoglobin A1c	6.0	<5.7	%
Urinalysis	(+) trace occult blood, (-) protein, (-) WBC	-	-

**Figure 2:**
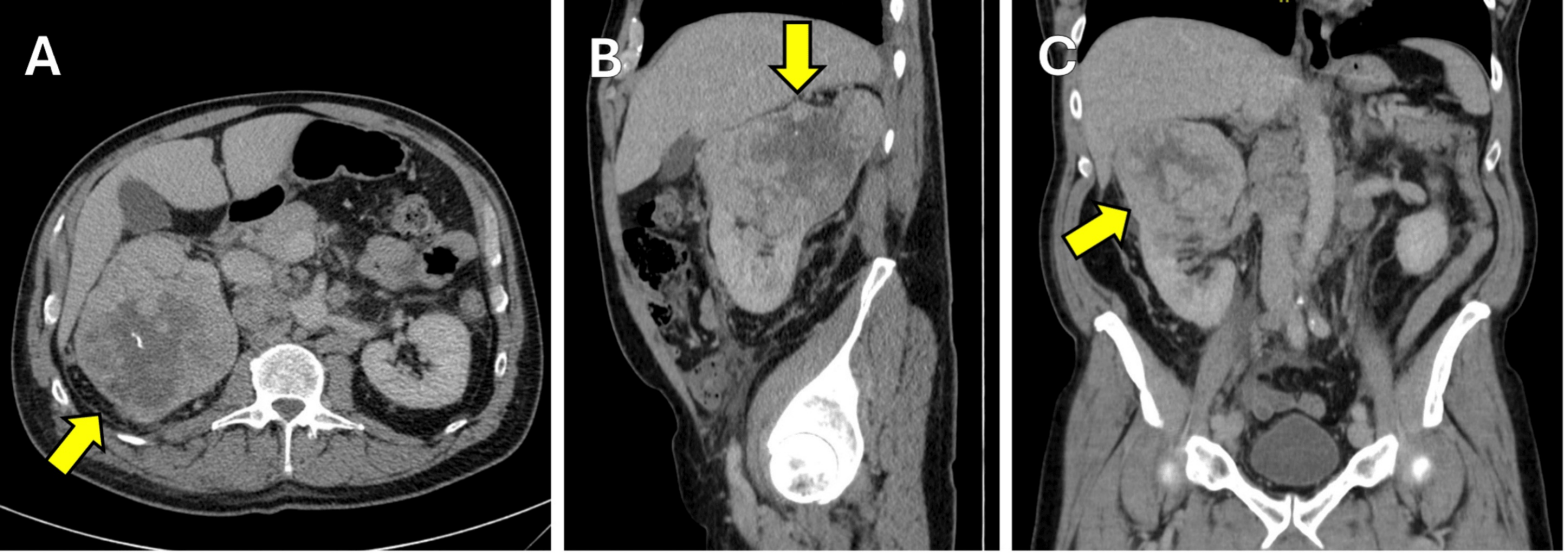
(A) Axial, (B) sagittal, and (C) coronal views of right renal mass.

Cross-sectional imaging was obtained, demonstrating an 11.7 cm mass in the right kidney and nodules in the right retroperitoneum consistent with metastatic RCC (Figure 2). The patient was referred to Oncology. Core biopsy of the left para-aortic lymph node was then obtained; pathology showed clear cell carcinoma. The patient was treated with 10 cycles of neoadjuvant nivolumab (a PD-1 inhibitor) and cabozantinib (a tyrosine kinase inhibitor) prior to right radical nephrectomy.

The patient continued adjuvant nivolumab and cabozantinib monthly after nephrectomy, and his eyelash growth slowed significantly on treatment. Within one year, the patient experienced disease progression with progressive multi-station lymphadenopathy demonstrated on PET/CT. Nineteen months after his initial diagnosis, he passed away due to acute thrombotic complications of the carcinoma, including a large superior mesenteric vein thrombus. Autopsy demonstrated widely metastatic disease throughout the abdomen, lymph nodes, lungs, and myocardium.

## Discussion

Hypertrichosis is classically associated with endocrine disorders and medications, but has been reported as a rare paraneoplastic phenomenon of carcinomas [8,9]. The biochemical mechanism promoting hair growth remains poorly understood [8].

Eyelash trichomegaly is a specific type of hypertrichosis. The term "trichomegaly" was first coined by Gray in 1944, in reference to long "movie lashes" desired by Hollywood actresses [10]. Since that time, scientists have elucidated the basic pathophysiology of lash hair follicles. Over a span of 4-11 months, eyelashes cycle through phases of continuous growth (anagen phase), transition (catagen phase), and rest (telogen phase) [11]. Trichomegaly stems from the prolongation of the anagen phase, but the cause for the induction of this prolongation remains murky. In Oncology literature, epidermal growth factor receptor (EGFR) antagonists (i.e., cetuximab or erlotinib) have been strongly associated with trichomegaly [12,13], suggesting EGFR-mediated signaling may serve as a “molecular brake” on eyelash growth [14].

Trichomegaly was initially postulated to be a cutaneous manifestation specific to HIV/AIDS [15], but has since been reported in association with atopic conditions, congenital disorders, malnutrition, and malignancy [16]. Documented association with renal cancer is exceedingly rare. There exists one case report of a 55-year-old man who developed trichomegaly 13 months after radical nephrectomy for stage one renal adenocarcinoma; he was ultimately found to have recurrence of disease in the lymph nodes and died shortly after [17].

In the case of the current patient, the lengthening and thickening of his eyelashes and existing body hair differed from the diffuse lanugo that has been described in paraneoplastic hypertrichosis lanuginosa [9]. However, his presentation may have been a variant of this syndrome.

The clinical implications of RCC-related PNS are not well-established. PNS itself lacks a clear definition, but it is loosely described as a set of signs and symptoms triggered by a malignant tumor that cannot be associated with its primary site or metastases [18]. In a retrospective study of 2865 patients with localized RCC, those with PNS (defined as anemia, polycythemia, hypercalcemia, and liver dysfunction) had a higher risk of death from all causes (hazard ratio (HR) = 1.64; 95%CI = 1.35-1.99) and death from RCC (HR = 1.86; 95%CI = 1.19-2.92) [19]. These findings are difficult to extrapolate to patients in the metastatic setting and those with a more esoteric PNS, such as hypertrichosis.

Regardless, early detection of RCC by any sign or symptom significantly impacts patient outcomes. In the United States from 2014-2020, the five-year relative survival rate for patients with localized renal cancer was 93.3% (95%CI 92.9-93.6). This is compared to a five-year relative survival of only 18.2% (95%CI 17.5-18.9) for those with distant metastases [20]. Overall median survival for patients with metastatic RCC is 22 months (95%CI 20.2-26.5), though this has improved significantly with the advent of modern immune checkpoint and tyrosine kinase inhibitors [6,21].

## Conclusions

This case highlights the importance of pausing to consider more insidious etiologies when faced with an unusual mucocutaneous finding. Occult malignancy should be considered in patients who develop atypical hair growth patterns, especially those of advanced age. Further investigation is needed to elucidate the immunomodulatory mechanisms by which RCC and other cancers trigger the development of trichomegaly.
